# Shallowing of the bicipital groove as a sequela of long head biceps tendon tear

**DOI:** 10.1007/s00256-025-05007-z

**Published:** 2025-08-18

**Authors:** Michael K. Hoy, Robert C. Hoy, William B. Morrison, Sarah I. Kamel

**Affiliations:** 1 Division of Musculoskeletal Radiology, Department of Radiology, Thomas Jefferson University Hospital, 132 S. 10Th St. Suite 1079a, Philadelphia, PA 19107 USA; 2 Department of Orthopaedic Surgery, Temple University Hospital, 3401 N. Broad St, Philadelphia, PA 19140 USA

**Keywords:** Biceps brachii, Shoulder joint, Tendon injuries, Magnetic resonance imaging, Bicipital groove

## Abstract

**Objective:**

To evaluate the relationship of depth of the bicipital groove at the shoulder and chronicity of long head biceps tendon tear. To assess the effect of long head biceps tendon (LHBT) tears on the depth of the bicipital groove over time on shoulder MRI.

**Materials and methods:**

A retrospective study was conducted in two cohorts. The first cohort included age, gender, and BMI-matched patients with either a complete LHBT tear or an intact tendon on a single shoulder MRI. Bicipital groove depth was compared between these two groups. The second cohort consisted of patients with a complete LHBT tear and one or more follow-up shoulder MRIs, used to evaluate longitudinal changes in groove depth.

**Results and conclusion:**

In the first cohort (*n* = 80; 40 complete LHBT tears, 40 intact tendons), the average bicipital groove depth was significantly shallower in the tear group (3.38 ± 1.23 mm) compared to the intact group (5.33 ± 0.87 mm) (*p* < 0.0001). In the second cohort (*n* = 31), initial groove depth averaged 4.94 ± 1.37 mm, decreasing to 2.47 ± 1.44 mm on follow-up MRI at an average of 40 months. The findings suggest that the morphology of the bicipital groove is maintained by the presence of an intact LHBT. Complete tears result in a gradual, measurable decrease in groove depth. Recognition of groove shallowing on MRI may serve as a secondary sign of chronic long head biceps tendon tear, aiding in diagnosis and assessment of chronicity.

## Introduction

The biceps brachii muscle consists of two proximal tendinous origins that connect it to the shoulder: the long head and the short head. The long head biceps tendon (LHBT) originates from the superior glenoid tubercle and courses intraarticularly to the bicipital groove, an indentation along the anterior proximal humerus between the greater and lesser tuberosities [1]. The groove houses the LHBT along with its synovial sheath [2]. In a cadaveric study, Wafae et al. found the average bicipital groove depth to be 4.0 mm, with a range of 2.2–6.3 mm. They found the depth of the groove to represent 9.1–29.5% of the depth of the humerus [3].

Rupture of the LHBT is the most common injury to the biceps muscle–tendon complex [4]. Tears typically occur at the proximal intra-articular LHBT, leading to distal tendon retraction into the arm. The transverse humeral ligament has been consistently described as the primary stabilizer of the biceps tendon in the bicipital groove, proposed as a band of tissue that covers the biceps tendon and keeps it within the groove. Recent studies have suggested that rather than a separate band of tissue, the transverse humeral ligament represents superficial collagen fibers from the subscapularis tendon that continue over the biceps tendon and insert onto the greater tuberosity, while deep fibers from the subscapularis tendon insert onto the lesser tuberosity just medial to the long head biceps tendon [5]. If there is a tear involving the subscapularis tendon, the LHBT may subluxate or dislocate medially from the groove. MRI is considered the optimum diagnostic imaging for LHBT instability and tears [6]. In the evaluation of complete biceps tendon tears, MRI has reported sensitivity and specificity ranging between 56–90% and 75–100%, respectively [7–9]. However, to date, no MRI signs have been tested to indicate the chronicity of LHBT tears.

It is unknown how the groove may change in response to LHBT pathology. Studies have investigated whether the morphology of the groove plays a role in propensity to develop LHBT pathology, but no correlation has been found [10].

The objective of this study was to evaluate changes in bicipital groove morphology associated with chronic LHBT tears on MRI. We aimed to determine whether loss of tendon integrity leads to measurable shallowing of the groove over time.

We hypothesize that in the absence of the LHBT, the bicipital groove progressively remodels, resulting in decreased depth that may be detectable on MRI and correlate with the chronicity of the tear.

## Materials and methods

### Patient selection

Institutional review board approval was obtained for this retrospective Health Insurance Portability and Accountability Act–compliant study, and the requirement for informed consent was waived due to the retrospective design of the study. The study was performed in accordance with the principles expressed in the Declaration of Helsinki. No funding was used for this study. A retrospective review of MRI shoulders performed between 2019 and 2020 was performed to identify patients with a complete LHBT tear reported. Imaging reports were mined for terminology and variations of the phrase “complete rupture/tear of the long head biceps tendon.” Patients with a surgical history of biceps tenodesis or other surgical changes in the shoulder were excluded from the study. Studies with disagreement between reviewers on the presence of complete LHBT were also excluded. An additional exclusion criterion included exams with artifact or poor quality that precluded accurate measurement of the groove. This yielded a study group entitled "complete LHBT tear". An age, gender, and BMI matched control group without biceps tendon or subscapularis tendon pathology was identified using the search term “intact/normal long head biceps tendon.”

**Fig. 1:**
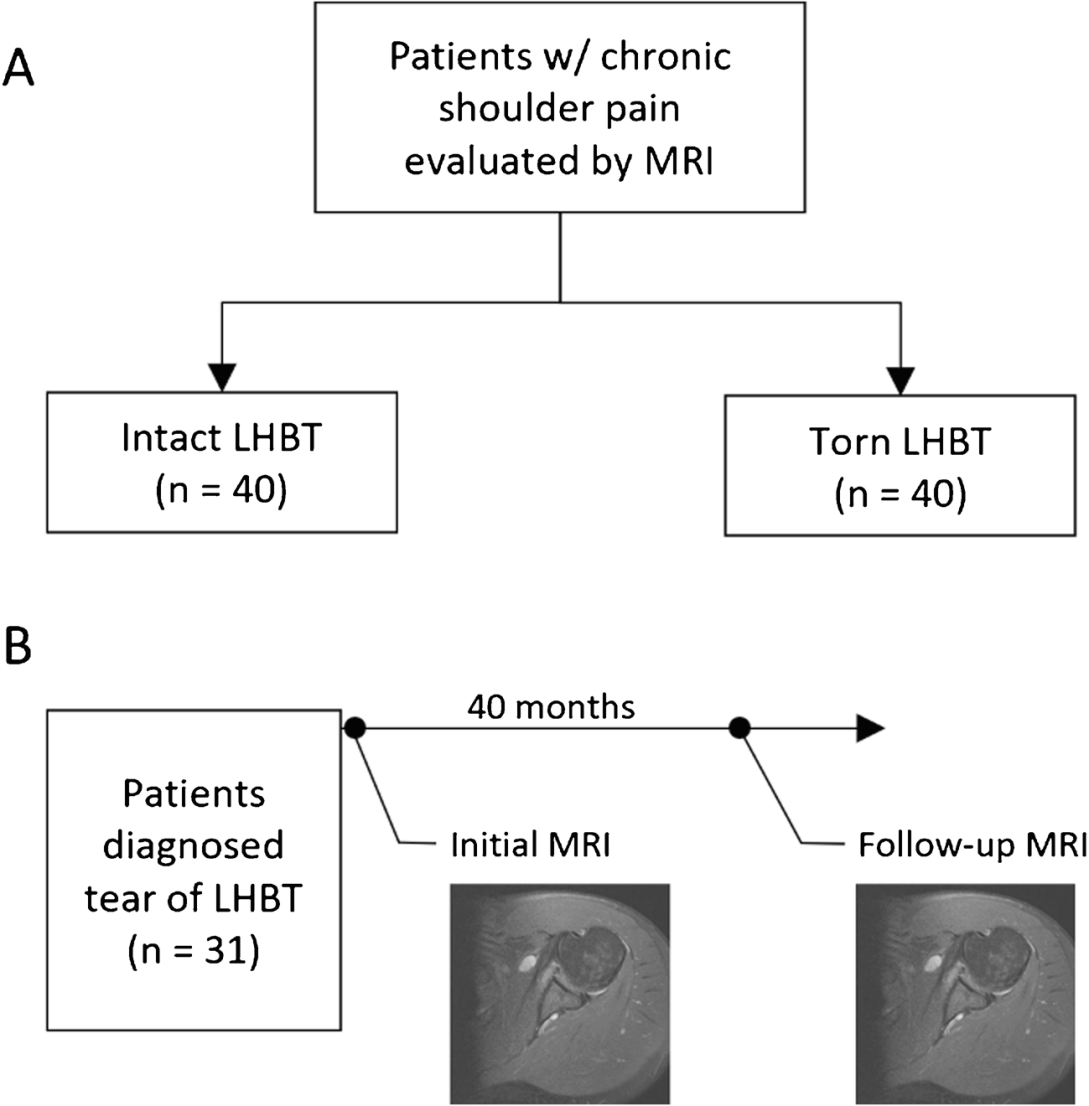
Study design detailing normal vs. complete LHBT tear MRI measurement as well as temporal measurement of groove (LHBT, long head biceps tendon). **A** Patient cohort with a single exam time point. **B** Patient cohort with access to follow-up exams

Additionally, a subset of patients was identified that had a single study with a complete LHBT tear and at least one follow-up shoulder MRI performed (Fig. 1). These studies were performed between 2014 and 2023.

### MR imaging parameters

All MR imaging examinations were performed with 1.5-T MR imaging systems (Optima and Signa; GE Medical Systems, Milwaukee, WI) and dedicated shoulder coils.

### Image analysis

**Fig. 2:**
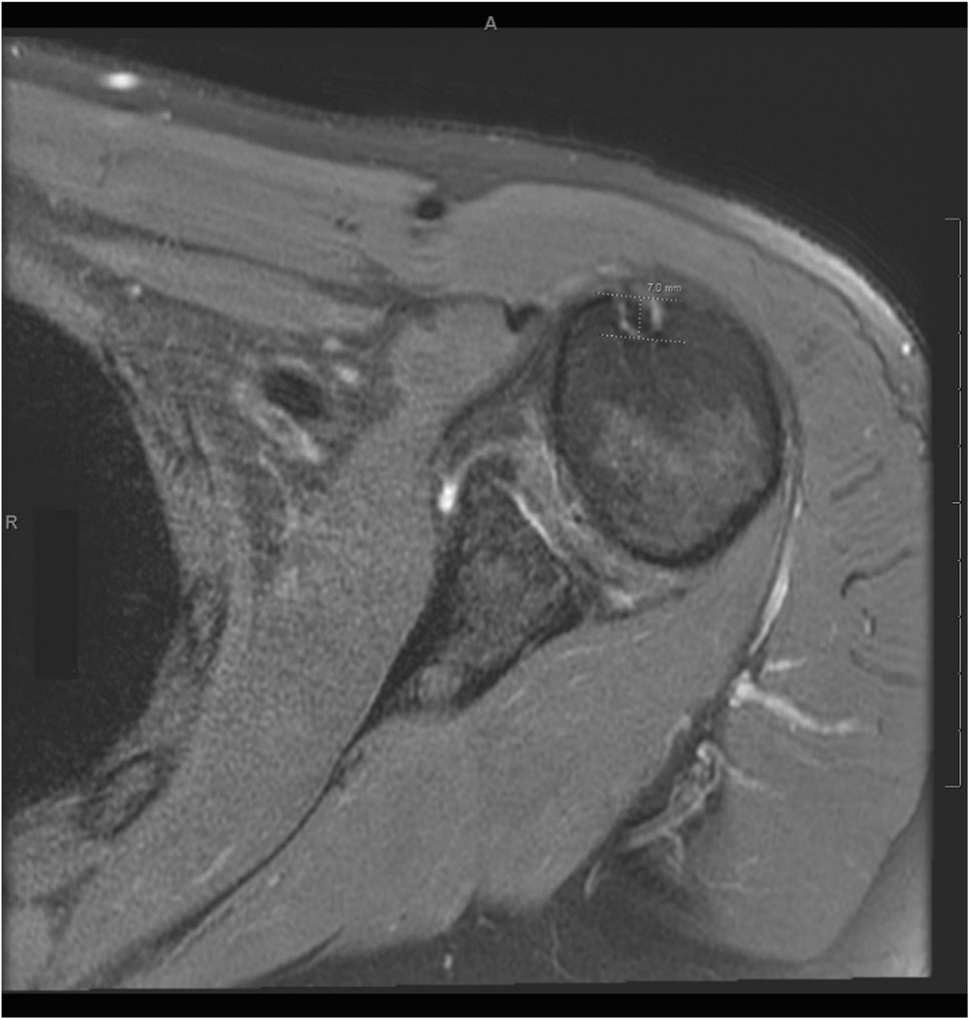
Measurement of biceps groove depth on axial MRI. A line was drawn from the greater tuberosity to the lesser tuberosity at the midportion of the groove. A parallel line was drawn at the deepest part of the groove, followed by a perpendicular line to measure the deepest part of the groove

Images were evaluated by two reviewers on a P.A.C.S. (Picture Archiving and Communication System) workstation to confirm the presence of a complete LHBT tear and depth of the bicipital groove, with independent measurements averaged. Bicipital groove morphology was assessed using axial MRI images, following the technique described by Abboud et al. [10, 11] (Fig. 2). Measurements were performed on proton density axial slices through the mid portion of the proximal humerus, typically between the 4th and 6th most cephalad images. The depth of the bicipital groove was measured by drawing a line connecting the peaks of the greater and lesser tuberosities, drawing a parallel line at the deepest part of the groove, and then measuring the perpendicular distance between the lines. Each measurement was performed on the deepest portion of the groove and repeated independently by two reviewers to ensure reproducibility.

### Measurement reproducibility/statistical analysis

Two fellowship-trained musculoskeletal radiologists with 6 years and 9 years of experience independently reviewed all imaging studies, blinded to clinical history, prior imaging, or the original study interpretation. Prior to the study, both readers underwent a calibration session using 10 cases outside the study dataset to standardize assessment criteria. All measurements were performed independently and blinded to each other’s assessments.

For the case–control study component, an independent *t*-test and chi-square test were used to compare demographic differences between the cases and control group. An intraclass correlation coefficient (ICC) was run using a two-way mixed model with absolute agreement to determine the intra-rater reliability of the measurements obtained by the raters at two time points for all patients (cases and controls). ICC values were interpreted as follows: < 0.50 poor, 0.50–0.75 moderate, 0.75–0.90 good, and > 0.90 excellent reproducibility. A two-way ANOVA was conducted to examine the effects of gender and BMI category on bicipital groove depth. An independent samples *t*-test was used to determine the differences in bicipital groove depth between cases and controls.

For the longitudinal study component, an ICC was run using a two-way mixed model using absolute agreement to determine reliability between each of the measurements obtained by the raters at the two time points. A Pearson’s correlation was used to assess the relationship between duration of LHBT tear and change in bicipital groove depth. For this analysis, the measurements provided by the two raters were first averaged for each of the time points, and the difference between the two measurements was used for the correlation. A linear regression was also performed to understand the effect of time following LHBT tear on the change in the groove depth. Statistical analysis was performed using Microsoft Excel Analysis ToolPak and GraphPad Prism® software (Boston, MA).

## Results

### Case–control component

For the case–control study component, 80 patients met inclusion criteria, and a summary of the two patient groups and their demographic characteristics can be seen in Table 1. There was no statistically significant difference in age, gender, or BMI between the two groups. There was excellent intra-rater reliability found for the measurements obtained for all patients, with an ICC of 0.96 (95% CI 0.97–0.98, *p* < 0.001).

**Table 1:** Case–control comparison of patients with complete LHBT tear vs. intact tendon

Parameter	LHBT tear group (*n* = 40)	Control group (*n* = 40)	*p*-value
Sex (M/F)	27/13	28/12	
Average age (years)	66 ± 13.0	61 ± 11.2	< 0.0001
Average BMI (kg/m^2^)	28.0 ± 6.2	28.8 ± 4.3	< 0.0001
Bicipital groove depth (mm)	3.38 ± 1.23	5.33 ± 0.88	< 0.0001

Groups were matched for age, gender, and BMI. Statistical comparison was performed using an independent *t*-test for continuous variables

*LHBT* long head of biceps tendon

There was no statistically significant interaction between gender and BMI on bicipital groove depth (*p* = 0.21). Mean bicipital groove depth was higher in controls (5.33 ± 0.8 mm) than cases (3.38 ± 1.2 mm), which was statistically significant (*p* < 0.001) (Table 1).

### Longitudinal component

For the longitudinal study component, 31 patients were included with a mean age of 62 ± 9.9 years (range 49–78) and 11 males (61%). The mean time interval between studies was 40 ± 48 months (range 14–183). A high degree of reliability was found for the bicipital groove depth measurements on the initial scan with an ICC of 0.85 (95% CI 0.61–0.94, *p* < 0.001). Moderate reliability between raters was found on the subsequent scan with an ICC of 0.668 (95% CI 0.56–0.87, *p* = 0.003).

There was a strong positive correlation between the time interval between MRI examinations and the decrease in bicipital groove depth (*r* = 0.575, *p* = 0.013). A linear regression established that the time interval following LHBT tear could statistically significantly predict the shallowing of the groove F(1,16) = 7.89, *p* = 0.01 using the following equation: Change in groove depth (mm) =  − 2.097 + (− 0.018 × time in months).

Initial bicipital groove depth measured 4.94 ± 1.37 mm (range 3–8 mm). At follow-up, the mean depth had decreased to 2.47 ± 1.44 mm (range 1–5 mm), representing a statistically significant reduction (*p* < 0.0001) (Table 2).

**Table 2:** Longitudinal cohort with complete LHBT tear and follow-up

Parameter	Value	*p*-value
Total patients	31	
Sex		
Male	22	
Female	11	
Average age (years)	62 (range 49–78)	
Length of follow-up (months)	40 (range 14–180)	
Bicipital groove depth (mm)		
Initial	4.94 ± 1.37	
Follow-up	2.47 ± 1.44	< 0.0001

Statistical comparison performed using paired *t*-test (significance threshold, *p* < 0.05)

*LHBT* long head of biceps tendon

## Discussion

**Fig. 3:**
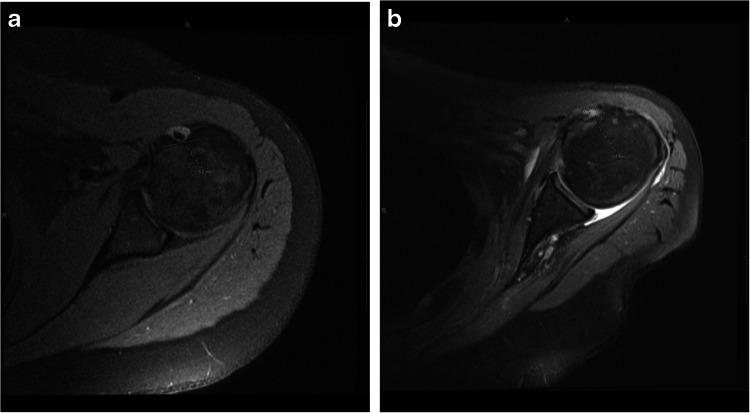
Axial MR images of normal LHBT and chronically torn LHBT. **A** A 53-year-old woman with an intact long head biceps tendon in the bicipital groove, with groove depth measuring 5.0 mm. **B** A 59-year-old woman with a complete tear of the long head biceps tendon, with shallowing of the bicipital groove, demonstrating a groove depth of 3.1 mm

The results of our study suggest that the depth of the bicipital groove is dependent on the integrity of the LHBT. Patients with a complete LHBT tear were found to have a shallower groove when compared to those without a tear (Fig. 3). Longitudinal imaging in those with complete LHBT tear revealed groove shallowing over time. These findings suggest that the depth of the bicipital groove can be used to determine the chronicity of complete LHBT tear: a deep groove indicating a recent tear and a shallow groove indicating the tear is more likely chronic.

Shallowing of the bicipital groove following a complete LHBT tear may reflect adaptive osseous changes secondary to altered biomechanical loading. With an intact tendon, the LHBT exerts mechanical forces on the bicipital groove as it transmits loads during shoulder motion. These compressive and tensile forces contribute to the maintenance of cortical bone along the groove. Following a complete LHBT tear, this mechanical stimulus is lost, leading to osseous remodeling through osteoclast and osteoblast activity. Over time, this results in measurable groove shallowing in response to the decreased force, consistent with Wolff’s law that bone tissue forms and is remodeled in response to the mechanical forces it experiences [12].

This process may also involve histological changes at the bone-tendon interface, dependent on the presence of a tendon in the groove. Previous studies have demonstrated that fibrocartilage is present within the bicipital groove, which may function as an adaptation to the compressive loading [13, 14]. In the setting of complete LHBT tear, this fibrocartilaginous lining has been shown to regress and be replaced by loose connective tissue that resembles synovium, which further supports the concept of structural remodeling in response to loss of mechanical load [14].

**Fig. 4:**
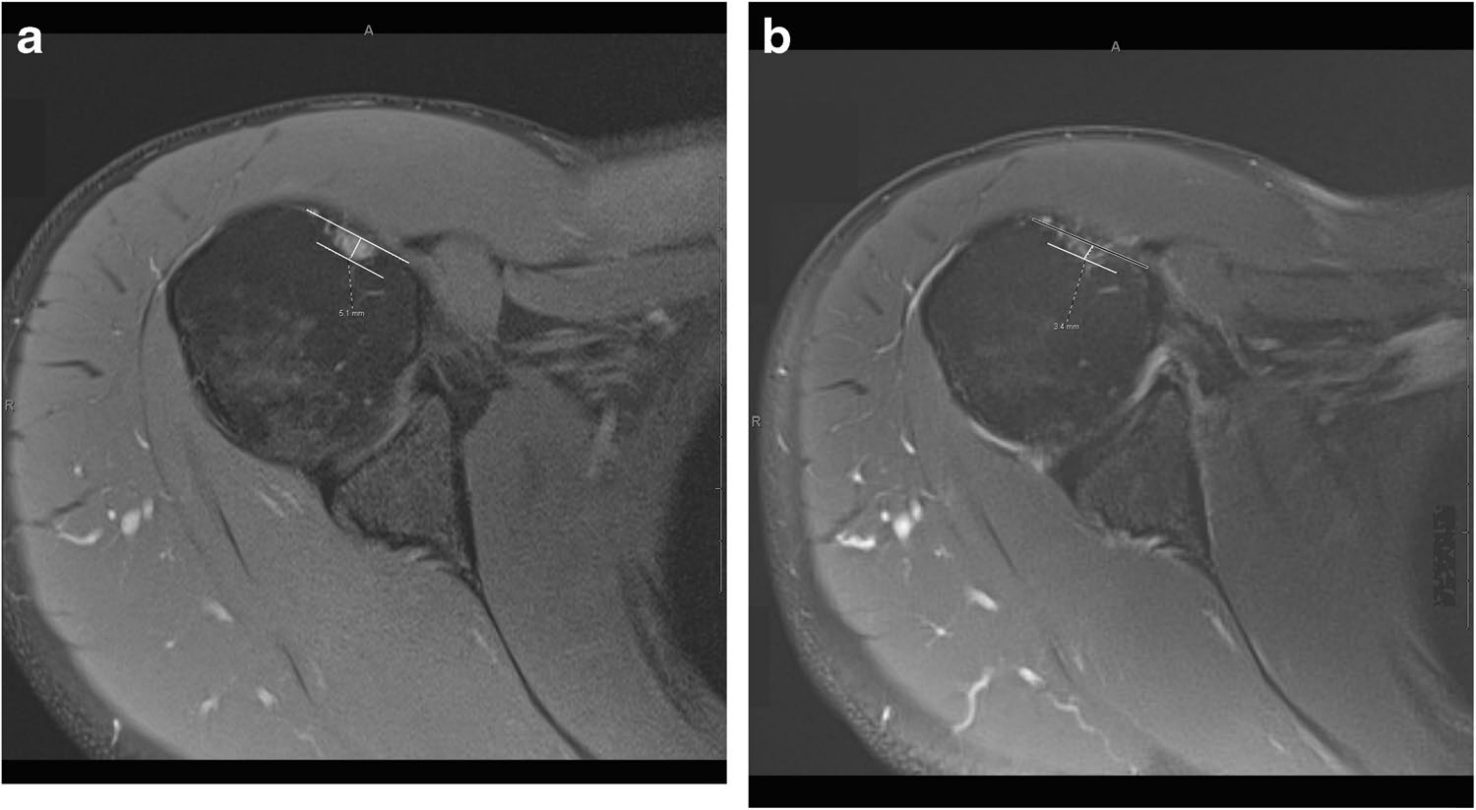
Shallowing of the bicipital groove after complete tear of the long head biceps tendon. **A** A 57-year-old man with a complete tear of the long head biceps tendon with groove depth measuring 5.1 mm. **B** Follow-up examination 14 months later demonstrates shallowing of the groove, with groove depth measuring 3.4 mm

Multiple prior studies have debated the interdependence of the biceps groove and LHBT tendinopathy. Pfahler et al. were among the first to report a significant correlation between biceps groove morphology and LHBT pathology. With ultrasonographic examination of the biceps tendon, they observed that a flattened bicipital groove was associated with LHBT pathological changes measured on a 0–10 point scale [15]. In a prospective study of 75 consecutive patients, Abboud et al. utilized direct arthroscopic visualization to classify LHBT pathology into normal, inflamed, partially torn, or ruptured. They then measured bicipital groove morphology on MRI and demonstrated no significant correlation of LHBT pathology with bicipital groove total opening angle, medial wall angle, or depth [10]. However, only 13 patients with ruptured LHBT were included in the study of 75 total patients, limiting the power of evaluating the correlation between biceps groove depth and complete LHBT tears. Shah et al. evaluated the arthroscopic findings following patients with subscapularis tendon tears with and without associated biceps tendon pathology. In their study, five patients had complete tears and 44 patients had incomplete lesions. They observed a decreased depth of the bicipital groove in patients with subscapularis tendon tears and biceps tendon pathology [16]. Most recently, Cardoso et al. published a series of papers which examined bicipital groove depth, width, and cross-sectional area (CSA) on both radiographs and ultrasound. They correlated these measurements to LHBT pathology examined during arthroscopy. LHBT pathology was classified as tendinopathy, partial disruption, and complete tear. When comparing patients with normal and abnormal LHBT, they found no difference in bicipital groove width, depth, or CSA [17]. Their study categorized all forms of LHBT pathology—including tendinopathy, partial-thickness tears (< 50% and > 50%), and complete tears—under a single “abnormal LHBT” group. Notably, only four cases in their cohort represented complete LHBT tears. This approach may have limited their ability to detect morphological changes specific to complete tendon rupture. The present study supports the correlation between decreased depth of the bicipital groove and biceps tendon tears, as well as demonstrates the use of MRI to determine chronicity of a LHBT tear based on groove characteristics. Notably, the present study includes only patients with a complete tear to the LHBT in the abnormal group. This suggests that changes to the biceps groove occur when the LHBT is absent (Fig. 4).

Previous authors have hypothesized anatomic morphology of the bicipital groove and proximal humerus may predispose patients to LHBT tears, with anatomically shallow and/or narrow grooves leading to tendon damage [15–19]. However, a temporal relationship in which LHBT pathology leads to changes in bicipital groove morphology had not been previously established. Here, we report on this temporal relationship and demonstrate that complete tear of the LHBT leads to a shallowing of the groove over time. Our findings suggest that a shallow groove is more likely a sequela of injury, not a predisposing factor to injury.

The clinical implications of the shallowing of the groove to the surrounding structures remain unknown. In patients who present with acute (< 12 week) rupture of the LHBT, the incidence of supraspinatus or subscapularis tendon tearing is significantly increased (incidence, 85–93%) [20, 21]. Rupture of the LHBT may lead to morphological changes to the proximal humerus, which result in biomechanical changes that compromise the rotator cuff structure. Alternatively, a compromised rotator cuff may increase stress on the LHBT and lead to its failure. Additional research is warranted to investigate if the morphological changes to the bicipital groove observed in this study play a role in the interdependence of the LHBT and the rotator cuff.

To date, there are no reliable imaging methods to evaluate the chronicity of a LHBT tear. The progressive shallowing of the biceps groove in response to a complete LHBT tear is a potential opportunity to assess the chronicity of an LHBT tear. In this study, even the shortest interval between initial and follow-up MRI of 14 months displayed a measurable decrease in bicipital groove depth (5 to 3 mm). Further evidence is required to evaluate the temporal progression of this response and determine its utility in determining the chronicity of an LHBT tear. Furthermore, ultrasound has been previously demonstrated to be an accurate and reliable method for the measurement of the bicipital groove depth [17, 22]. Ultrasound could serve as a less costly option in determining the chronicity of a tear in patients who present with suspected biceps pathology.

### Limitations

This study has several limitations that should be considered when interpreting the findings. The retrospective design limits control over imaging follow-up intervals and introduces variability with the timing of post-tear assessments. A prospective study with standardized imaging intervals would allow for more precise evaluation of temporal changes in bicipital groove morphology. The relatively small sample size of the longitudinal cohort with complete LHBT tears and follow-up imaging represents an additional limitation. While the observed trend toward groove shallowing was consistent across cases, the small number of patients limits the generalizability of the findings and restricts the ability to perform subgroup analyses. While a standardized measurement technique was employed, reviewers were not blinded to the presence of a biceps tendon tear, which may introduce measurement bias. Technical factors pose additional limitations. Variability in the exact location of groove depth measurement and differences in patient positioning during MRI acquisition may affect the accuracy and reproducibility of measurements. Slice thickness of 4 mm results in variation of where the measurement is performed on an axial sequence. Additionally, the use of proton density (PD)–weighted images rather than T1-weighted sequences reduces the reliability of cortical bone delineation, potentially impacting measurement precision. Imaging performed across multiple sites within the same institution contributes to variability in image quality and acquisition parameters. The absence of clinical data, such as surgical correlation of symptom duration, also limits the ability to correlate imaging findings with clinical severity or chronicity. Rotator cuff tears may independently influence the morphology of the bicipital groove and confound the observed association with LHBT tears, specifically with tearing of the subscapularis tendon. An additional limitation is the absence of longitudinal imaging in patients with initially intact LHBT who subsequently sustained a complete tear. Pre- and post-tear comparison within the same individuals would provide additional evidence to support the hypothesis that a complete LHBT tear leads to progressive shallowing of the bicipital groove. This represents a potential avenue for future investigation. Overall, larger prospective studies are needed to validate these observations and further characterize the temporal progression of osseous remodeling. Despite these limitations, the study provides evidence of a measurable association between complete LHBT tears and progressive shallowing of the bicipital groove, which may have diagnostic implications in assessing chronicity.

## Data Availability

Data used in this study is available upon request.
